# Diabetes among women with preterm births: outcomes of a Brazilian multicenter study

**DOI:** 10.31744/einstein_journal/2023AO0515

**Published:** 2023-12-06

**Authors:** Juliana da Costa Santos, José Paulo Siqueira Guida, Christopher Cralcev, Tabata Zumpano Dias, Renato Passini, Giuliane Jesus Lajos, Rodolfo Carvalho Pacagnella, Ricardo Porto Tedesco, Marcelo Luis Nomura, Patricia Moretti Rehder, José Guilherme Cecatti, Maria Laura Costa

**Affiliations:** 1 Universidade Estadual de Campinas Campinas SP Brazil Universidade Estadual de Campinas , Campinas , SP , Brazil .

**Keywords:** Hypertension, pregnancy-induced, Preterm birth, Gestational diabetes, Maternal mortality

## Abstract

**Objective:**

The objective was to compare the maternal and perinatal characteristics and outcomes between women with and without diabetes in a Brazilian cohort of women with preterm births.

**Methods:**

This was an ancillary analysis of the Brazilian Multicenter Study on Preterm Birth, which included 4,150 preterm births. This analysis divided preterm births into two groups according to the presence of diabetes; pregestational and gestational diabetes were clustered in the same Diabetes Group. Differences between both groups were assessed using χ ^2^ or Student’s *t* tests.

**Results:**

Preterm births of 133 and 4,017 women with and without diabetes, respectively, were included. The prevalence of diabetes was 3.2%. Pregnant women aged ≥35 years were more common in the Diabetes Group (31.6% *versus* 14.0% non-diabetic women, respectively). The rate of cesarean section among patients with diabetes was 68.2% *versus* 52.3% in non-diabetic cases), with a gestational age at birth between 34 and 36 weeks in 78.9% of the cases and 62.1% of the controls. Large-for-gestational-age babies were 7 times more common in the Diabetes Group.

**Conclusion:**

Preterm birth among Brazilian women with diabetes was more than twice as prevalent; these women were older and had regular late preterm deliveries, usually by cesarean section. They also had a greater frequency of fetal morbidities, such as malformations and polyhydramnios, and a higher proportion of large-for-gestational-age and macrosomic neonates.

## Summary

In Brief

In a cohort of Brazilian pregnant women who had preterm births, the diabetic pregnant women were 35 years or older and had greater rates of provider-initiated preterm births, cesarean sections, and late preterm deliveries resulting in large-forgestational- age babies with more neonatal hypoglycemia.

## Abstract (key points)

Highlights

Diabetes prevalence at 3.2% among women who had preterm births in Brazil.

A۪۪lmost half of the preterm births of pregnant women with diabetes were therapeutic.

Predominance of late preterm cesarean sections with large-for-gestational-age babies.

## Graphical abstract

**Figure:**
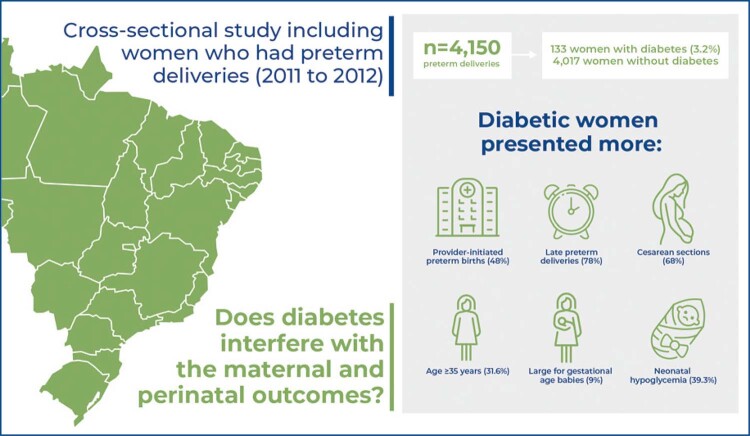

## INTRODUCTION

*Diabetes mellitus* is a health condition of increasing concern globally. Data from the 2016 World Health Organization’s Global Report on Diabetes estimated that 422 million adults have diabetes, a prevalence that has nearly doubled since 1980 ^( 1 )^ reflecting an increase due to associated risk factors, such as obesity.

Diabetes is a group of metabolic disturbances that can be classified according to its etiopathogenesis into type 1 diabetes (destruction of pancreatic beta cells), type 2 diabetes (reduced insulin secretion and insulin resistance), and gestational diabetes (glycemic control disturbances induced by pregnancy that cannot be balanced due to an impairment in insulin secretion). ^( 2 )^

Currently, it is estimated that one in six births occur in pregnant women presenting with hyperglycemia, and 84% of these cases represent gestational *diabetes mellitus* cases. ^( 2 )^ These cases are associated with adverse maternal and perinatal outcomes, including fetal macrosomia, congenital malformations, perinatal death, fetal growth restriction, and preterm birth (PTB). ^( 3 )^ Furthermore, recent studies have investigated the long-term consequences of hyperglycemia in the offspring of diabetic mothers, with evidence suggesting an increase in metabolic disturbances, such as metabolic syndrome in adult life and epigenetic reprogramming. ^( 4 - 6 )^ Considering the possible negative outcomes of diabetes during pregnancy, PTB could increase the aforementioned neonatal complications and long-term consequences.

Preterm birth is a leading cause of neonatal morbidity and mortality worldwide, defined as birth before the 37 ^th^ week of pregnancy. It can be classified as spontaneous or provider-initiated, and the latter occurs when maternal and/or fetal complications indicate medical intervention to anticipate birth. ^( 7 )^

This analysis aimed to compare the impact of diabetes in a population of women with preterm delivery.

## OBJECTIVE

To compare the maternal and perinatal characteristics and outcomes in patients with and without diabetes, we obtained data from the Brazilian Multicenter Study on Preterm Birth.

## METHODS

This is an ancillary analysis of the Brazilian Multicenter Study on Preterm Birth (EMIP - *Estudo Multicêntrico de Investigação de Prematuridade* ). This cross-sectional multicenter study assessed women who had PTB in 20 obstetric referral hospitals in three regions of Brazil between April 2011 and July 2012. The research protocol and main results of this cohort study have been published elsewhere. ^( 8 , 9 )^ Briefly, the EMIP included all women with PTB in the participating facilities during the study period who provided informed consent. Medical charts were reviewed, and data on maternal and perinatal outcomes were obtained and stored on a web-based platform hosted on a server from the coordinating institution.

For the present analysis, all PTB cases were included, and women were divided into two groups according to the reported occurrence of diabetes. All women with pre-gestational diabetes and those diagnosed with gestational diabetes were included in the Diabetes Group, whereas women with no diagnosis of diabetes were included in the Non-diabetes Group.

We applied pragmatic criteria for diabetes based on data obtained from medical charts. Women were included in the Diabetes Group when the data collection chart described diabetes as a maternal disease prior to pregnancy, or as a chronic disease or complication of pregnancy. No information was available regarding the treatment of diabetes during pregnancy. Delivery was considered preterm if it occurred before 37 weeks of gestation.

The main variables considered were maternal age, marital status, morbidity history, antenatal care, number of prenatal visits, initial and final body mass index, vulvovaginitis during pregnancy, fetal morbidities, onset of labor, route of delivery, amniotic fluid disorders, gestational age at birth, birth weight adequacy to gestational age, need for orotracheal intubation at birth, average length of hospital stay, and neonatal hypoglycemia.

Statistical analysis was performed using version 22.0.0.0 of SPSS software. Frequencies of outcomes were obtained in each group and compared using the χ ^2^ or Fisher’s (if a cell count was less than 5), and Student’s *t* tests for bivariate analysis of categorical and numeric variables, respectively. Statistical significance was set at p<0.05.

The original study was approved by the Institutional Review Board of the *Universidade Estadual de Campinas* (approval number 704/2009) and the local Institutional Review Boards of each hospital where the data were collected. Informed consent was obtained from all participants.

## RESULTS

**Figure 1:**
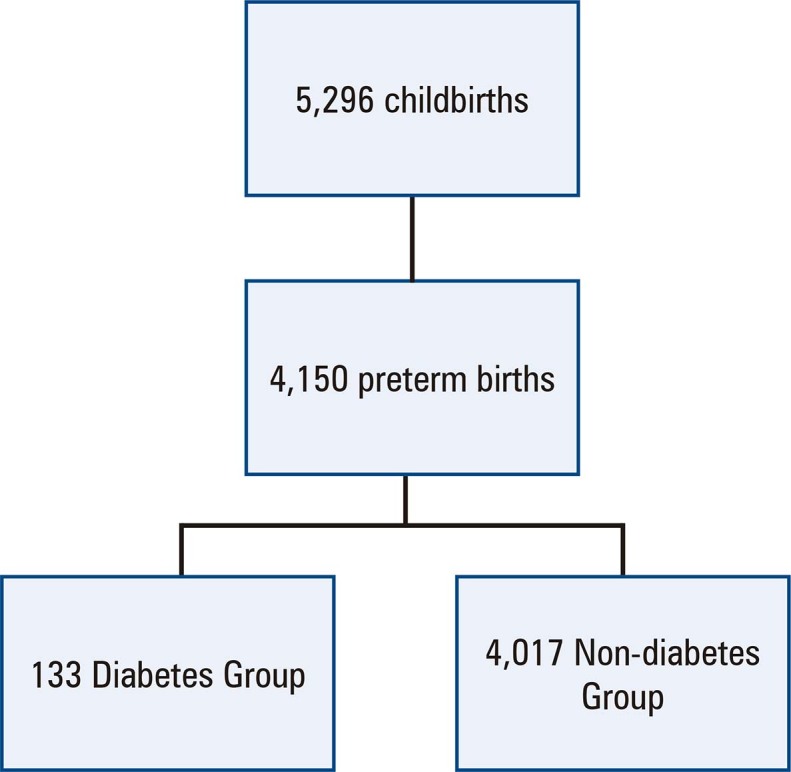
Flowchart of inclusion

A total of 4,150 PTB cases were included; among them, 133 (3.2%) were classified as diabetic and 4,017 (96.8%) as non-diabetic. Figure 1 shows this study’s inclusion flowchart. In the Diabetes Group, 50 women were diagnosed prior to pregnancy, and 83 were diagnosed during pregnancy.

The social and demographic characteristics are presented in table 1 . PTBs were more frequent in older women with diabetes than in those without diabetes (maternal age ≥35 years; 31.6% *versus* 14.0%; p<0.01). Preterm birth occurred less frequently in women with diabetes without a partner (15.0% *versus* 23.3%, p=0.03), and there was a higher frequency of preterm delivery among women with diabetes with previous medical conditions than in the Non-diabetic Group (52.6% *versus* 24.9%; p<0.01). There were no differences in ethnicity, level of education, previous PTB, or prevalence of chronic hypertension.

**Table 1:** Maternal social and demographic characteristics, and clinical and obstetric background of women with preterm birth, according to the diagnosis of diabetes

Maternal characteristics	Preterm births		

	Diabetic Group (n=133)	Non-diabetic Group (n=4,017)	p value
Maternal age (years)			<0.01 ^†^
≤19	7 (5.3)	857 (21.3)	
20–34	84 (63.2)	2,596 (64.6)	
≥35	42 (31.6)	563 (14.0)	
Skin color			0.69
White	61 (45.9)	1,772 (44.1)	
Other	72 (54.1)	2,245 (55.9)	
Marital status			0.03 ^†^
With partner	113 (85.0)	3,082 (76.7)	
No partner	20 (15.0)	935 (23.3)	
Schooling (years)			0.34
≤8	57 (43.5)	1,584 (40.1)	
9-12	60 (45.8)	2,045 (51.7)	
>12	14 (10.7)	326 (8.2)	
Morbid history			<0.01 ^†^
Yes	70 (52.6)	1,001 (24.9)	
No	63 (47.4)	3,014 (75.1)	
Chronic hypertension			0.54
Yes	10 (7.5)	364 (9.1)	
No	123 (92.5)	3,651 (90.9)	
Previous therapeutic preterm birth			0.61
Yes	13 (9.8)	339 (8.5)	
No	120 (90.2)	3,642 (91.5)	
Previous preterm birth			0.26
Yes	31 (23.5)	781 (19.5)	
No	101 (76.5)	3,225 (80.5)	
Previous low birthweight newborn*			0.54
Yes	25 (18.8)	667 (16.8)	
No	108 (81.2)	3,315 (83.2)	

* Birth weight lower than 2500g in any previous delivery; ^†^ p<0.05: statistically significant value.

Table 2 shows that all women included in the Diabetic Group received antenatal care. In the Non-diabetic Group, 3.8% did not have access to antenatal care at all (p=0.02). Gestational age at the onset of antenatal care was similar; however, most women with diabetes (70.8%) had at least six visits, against 53.2% among women without diabetes (p<0.01).

**Table 2:** Pregnancy outcomes and other characteristics of women with preterm birth according to the diagnosis of diabetes

Clinical evolution	Preterm births		

	Diabetic Group (n=133)	Non-diabetic Group (n=4,017)	p value
Antenatal care			0.02 ^†^
Yes	133 (100.0)	3,866 (96.2)	
No	0 (0)	151 (3.8)	
Onset of prenatal care			0.10
First trimester	66 (57.4)	2,121 (64.9)	
Second or third trimester	49 (42.6)	1,149 (35.1)	
Number of prenatal visits			<0.01 ^†^
Adequate (≥6)	85 (70.8)	1,832 (53.2)	
Inadequate (<6)	35 (29.2)	1,614 (46.8)	
Weight gain in pregnancy			0.26
≤7kg	35 (27.3)	1,164 (33.9)	
8-12kg	45 (35.2)	1,167 (33.9)	
>12kg	48 (37.5)	1,107 (32.2)	
Initial body mass index			<0.01 ^†^
<18.5kg/m ^2^ : underweight	4 (3.1)	298 (8.6)	
18.5–24.99kg/m ^2^ : normal	46 (36.2)	1,944 (56.2)	
≥25kg/m ^2^ : overweight/obesity	77 (60.6)	1,220 (35.2)	
Final body mass index			<0.01 ^†^
<18.5kg/m ^2^ : underweight	0 (0)	26 (0.8)	
18.5-24.99kg/m ^2^ : normal	14 (11.3)	975 (29.4)	
≥25kg/m ^2^ : overweight/obesity	110 (88.7)	2,314 (69.8)	
Paid work in pregnancy			0.04 ^†^
Yes	50 (79.4)	1,485 (88.2)	
No	13 (20.6)	199 (11.8)	
Smoking in pregnancy			0.08
Yes	12 (9.0)	579 (14.4)	
No	121 (91.0)	3,438 (85.6)	
Alcohol consumption in pregnancy			0.15
Yes (Frequently)	0 (0)	61 (1.5)	
No or seldom	132 (100)	3,928 (98.5)	
Anaemia			0.98
Yes	43 (33.3)	1,192 (33.2)	
No	86 (66.7)	2,395 (66.8)	
Vulvovaginitis in pregnancy			0.02 ^†^
Yes	37 (38.1)	660 (27.4)	
No	60 (61.9)	1,746 (72.6)	
Urinary tract infection during pregnancy			0.61
Yes	38 (31.7)	1,067 (33.9)	
No	82 (68.3)	2,079 (66.1)	
Dental inflammation/infection in pregnancy			0.41
Yes	26 (19.8)	680 (17.1)	
No	105 (80.2)	3,295 (82.9)	
Any other infection during pregnancy			0.21
Yes	5 (3.8)	260 (6.5)	
No	128 (96.2)	3,757 (93.5)	

^†^ p<0.05: statistically significant value. GA: gestational age.

Weight gain during pregnancy was similar in both groups; however, women with diabetes who were obese at the beginning or end of the antenatal care follow-up had more preterm deliveries. Alcohol consumption and smoking during pregnancy rates were low and similar in both groups. Approximately one-third of the women in both groups presented with anemia. Vulvovaginitis was significantly more frequent among women with diabetes (38.1% *versus* 27.4%, p=0.02), while the incidence of urinary tract, dental, or other infections was similar in both groups.

Considering other gestational complications, table 3 shows that polyhydramnios was present in 16.7% of the women with diabetes and 2.7% of the women without diabetes (p<0.01). The antenatal corticosteroid use was similar and low in both groups ( Table 3 ).

**Table 3:** Gestational characteristics of women with preterm birth according to the diagnosis of diabetes

Gestational characteristics	Preterm births		

	Diabetic Group (n=133)	Non-diabetic Group (n=4017)	p value
Fetal morbidity			<0.01 ^†^
Malformation	11 (8.5)	213 (5.8)	
Fetal growth restriction	10 (7.7)	373 (10.1)	
Other	19 (14.6)	264 (7.2)	
No	90 (69.2)	2,833 (76.9)	
Onset of labor			<0.01 ^†^
Spontaneous	55 (41.4)	2,162 (53.8)	
Induction of labor	16 (12.0)	558 (13.9)	
Planned cesarean section	62 (46.6)	1,297 (32.3)	
Route of labor			<0.01 ^†^
Vaginal	42 (31.8)	1,890 (47.7)	
Cesarean section	90 (68.2)	2,074 (52.3)	
Amniotic fluid disorders			<0.01 ^†^
Oligohydramnios	16 (12.1)	733 (19.8)	
Polyhydramnios	22 (16.7)	101 (2.7)	
No	94 (71.2)	2,859 (77.4)	
Antenatal corticosteroids use			0.56
Yes	44 (34.4)	1,401 (36.9)	
No	84 (65.6)	2,398 (63.1)	
Preterm birth classification			<0.01
Non-provider initiated preterm birth	69 (51.9)	2,613 (65.0)	
Provider initiated preterm birth	64 (48.1)	1,404 (35.0)	
Gestational age at birth			<0.01 ^†^
<34 weeks	28 (21.1)	1,521 (37.9)	
34–36 weeks	105 (78.9)	2,496 (62.1)	

^†^ p<0.05: statistically significant value.

Provider-initiated PTB was the main cause of PTB in the Diabetes Group, and its occurrence was more frequent in the Diabetes Group than in the Non-diabetes Group (48.1% *versus* 35.0%, p<0.001). In the Non-diabetic Group, spontaneous PTB was the main cause of premature birth (36.7%).

Late PTB (34–36 weeks) was more prevalent in both groups but was significantly more frequent (78.9%) among women with diabetes than in the non-diabetic group (62.1%) (p<0.01). Cesarean section was the most frequent route of delivery for PTB among women with diabetes (68.2%) compared to that in women without diabetes (52.3%) (p<0.01) ( Table 3 ).

The neonatal outcomes of preterm newborns among pregnant women with and without diabetes are presented in table 4 . Large-for-gestational-age newborns occurred more frequently in the Diabetic Group (9.1%) than in the Non-diabetic Group (1.2%), whereas the opposite was true for small-for-gestational-age babies (p<0.01). Furthermore, in the Diabetes Group, 4.5% of the women delivered newborns weighing >4,000g, whereas in the Control Group, 0.1% had the same outcome (p<0.01). Neonatal asphyxia (5 ^th^ minute Apgar score <7) and average length of stay in the neonatal intensive care unit were similar in both groups.

**Table 4:** Perinatal outcomes among women with preterm birth according to the diagnosis of diabetes

Perinatal results	Preterm births		

	Diabetic Group (n=133)	Non-diabetic Group (n=4,017)	p value
Birthweight adequacy to GA			<0.01 ^†^
Small for gestational age	16 (12.1)	1,066 (26.7)	
Adequate for gestational age	104 (78.8)	2,884 (72.1)	
Large for gestational age	12 (9.1)	47 (1.2)	
Birthweight			<0.01 ^†^
≤1500g	12 (9.1)	862 (21.6)	
1501 to 2500g	51 (38.6)	2,050 (51.3)	
>2500g and ≤4000g	63 (47.8)	1,080 (26.8)	
> 4000g	6 (4.5)	5 (0.1)	
5 ^th^ minute Apgar score <7			0.37
Yes	6 (4.7)	254 (6.7)	
No	122 (95.3)	3,554 (93.3)	
Orotracheal intubation at birth			0.02 ^†^
Yes	11 (8.9)	635 (16.7)	
No	113 (91.1)	3,159 (83.3)	
Surfactant use			0.08
Yes	9 (7.3)	604 (16.1)	
No	114 (92.7)	3,136 (83.9)	
Fetal malformation			0.10
Yes	19 (15.4)	405 (10.8)	
No	104 (84.6)	3,351 (89.2)	
Average length of hospital stay (days)	12.7	15.9	0.01 ^†^
Average length of NICU stay (days)	15.9	16.7	0.43
Ventilatory support			0.08
Yes	55 (44.0)	1,973 (52.0)	
No	70 (56.0)	1,823 (48.0)	
Any neonatal morbidity			0.43
Yes	84 (67.2)	2,672 (70.5)	
No	41 (32.8)	1,119 (29.5)	
Sepsis			0.10
Yes	17 (21.2)	759 (29.7)	
No	63 (78.8)	1,796 (70.3)	
Respiratory distress			0.11
Yes	56 (68.3)	2,010 (76.0)	
No	26 (31.7)	634 (24.0)	
Intraventricular hemorrhage			0.63
Yes	5 (7.7)	193 (9.5)	
No	60 (92.3)	1,846 (90.5)	
Neonatal hypoglycemia			<0.01 ^†^
Yes	33 (39.3)	582 (22.5)	
No	51 (60.7)	2,010 (77.5)	
Necrotizing enterocolitis			0.42
Yes	1 (1.2)	68 (2.6)	
No	82 (98.8)	2,517 (97.4)	
Pneumonia			0.35
Yes	3 (3.6)	158 (6.1)	
No	80 (96.4)	2,434 (93.9)	
Oxygen therapy at 28 days			0.36
Yes	5 (6.0)	228 (8.8)	
No	79 (94.0)	2,364 (91.2)	
Oxygen therapy at 56 days			0.68
Yes	2 (2.4)	81 (3.2)	
No	82 (97.6)	2,465 (96.8)	
Newborn’s condition at discharge or hospital transfer			0.23
Alive	117 (94.4)	3,430 (89.6)	
Dead	6 (4.8)	328 (8.6)	
No discharge*	1 (0.8)	70 (1.8)	
Newborn’s age (days) at death			0.75
≤7	4 (57.1)	223 (70.3)	
8-28	2 (28.6)	65 (20.5)	

* until 42 days after delivery; ^†^ p<0.05: statistically significant value. GA: gestational age; NICU: neonatal intensive care unit.

Table 4 presents adverse neonatal outcomes. Among these, orotracheal intubation at birth was more frequent in the Non-diabetic Group (8.9% *versus* 16.7%, p=0.02), and neonatal hypoglycemia was more frequent in the Diabetic Group (39.3% *versus* 22.5%, p<0.01). Ventilatory support, sepsis, respiratory distress, intraventricular hemorrhage, necrotizing enterocolitis, pneumonia, and oxygen therapy at 28 and 56 days of life were similar between groups. Death occurred in 4.8% of the diabetes-born children and 8.6% of the non-diabetes-born infants (p=0.23).

## DISCUSSION

This study aimed to assess the maternal and perinatal characteristics and outcomes of PTB in women with diabetes and compare them with those in the women without diabetes in a large Brazilian multicenter study. Maternal age ≥35 years, being overweight, and obesity were more frequent in women with diabetes. Polyhydramnios and vulvovaginitis were associated with diabetes in women with PTB. High cesarean section rates, a substantial number of large-for-gestational-age, and macrosomic newborns were also associated with diabetes in this sample.

However, these findings are not novel. According to a publication on the screening and diagnosis of *diabetes mellitus* in Brazil ^( 2 )^ the occurrence of hyperglycemia in pregnancy is facilitated by advanced maternal age, being overweight or obese, previous metabolic disorders, obstetric history of gestational diabetes, polyhydramnios, macrosomia, and fetal malformations in previous pregnancies.

According to the 2018 World Health Organization Global Status Report on Noncommunicable Diseases, obesity has an estimated prevalence of 26% among Brazilian women. ^( 10 )^ Pregnant women who are overweight and have obesity are at risk of developing complications such as diabetes during pregnancy and severe adverse maternal and perinatal outcomes. ^( 4 , 11 - 14 )^ Nonetheless, there is documentation of long-term repercussions in offspring exposed to diabetes in utero, which extend from neonatal complications to metabolic syndrome and epigenetic reprogramming. ^( 4 - 6 )^ As diabetes and obesity epidemics are rising, recent studies investigate “diabesity,” a phenomenon that could possibly be the greatest epidemic in human history. ^( 6 )^ Our sample presented an overall diabetes frequency of 3.2%, which was much lower than the global estimate of approximately 16%. ^( 2 )^ This could be a consequence of analyzing only PTB and/or underreported cases, considering that there was no procedure specifically directed at screening for this condition in the sample of women included in the study. Nevertheless, the results agree with most reported outcomes for diabetes and should raise attention to the relevance of this diagnosis and the follow-up of such women during pregnancy.

All pregnant women with diabetes received antenatal care, and some of them achieved an adequate number of six antenatal visits, according to the Brazilian Ministry of Health. ^( 15 )^ This could be explained by the early initiation of antenatal visits, motivated by the woman’s morbid history, or the need for more medical visits for metabolic control. However, the evaluation of the adequacy of the number of antenatal care visits has limitations among PTB cases; an inadequate number of visits could be just a consequence of the sample, which only includes PTB, and therefore reduces the possibility of a complete set of medical visits. Among the diabetic population studied, there were more late PTB (between 34 and 36 weeks GA) in the Diabetic Group than in the Non-diabetic Group.

The studied population replicated results observed in other studies and populations. At the beginning of pregnancy, half of the non-diabetic pregnant women had normal weight, with a smaller proportion being overweight and obese and even fewer underweight women. ^( 4 , 12 , 13 , 16 )^ However, most women with diabetes have been obese since the beginning of antenatal care. Obesity is a potentially treatable, noncommunicable disease that increases maternal risk during pregnancy, and a multidisciplinary team may introduce dietary and physical interventions to prevent obesity-related complications. ^( 17 , 18 )^

Diabetes is known to increase the risk of multiple adverse outcomes, such as hypertension, polyhydramnios, fetal growth disturbances (fetal growth restriction when vascular impairment is evident and fetal macrosomia when the fetus receives excessive nutrients), and fetal malformations. These defects usually occur in the heart, central nervous system, gastrointestinal tract, and the musculoskeletal and genitourinary tracts. ^( 4 , 12 - 14 )^ In this study, a higher prevalence of malformations was observed among pregnant women with diabetes.

Regarding the delivery route, metabolic control and the presence of maternal and/or fetal impairment must be considered. Therefore, higher rates of cesarean births in women with diabetes are more frequently reported in the literature among women who do not achieve satisfactory glycemic control. ^( 4 , 14 )^ Gestational age at birth was predominantly between 34 and 36 weeks, consistent with international data. ^( 11 )^

Women with diabetes delivered approximately two times more newborns weighing over 2500g, and seven times more infants were considered large for their gestational age. Half of the large-for-gestational-age infants in the Diabetes Group met the criteria for macrosomia (birth weight >4000g), with no correspondence in the Non-diabetic Group, which had a slight proportion of macrosomic newborns. Similar results have been reported in other studies. ^( 4 , 5 , 12 )^

The predominance of late PTB (between 34 and 36 weeks of gestational age) and greater birth weight could be some of the reasons why most cases did not present with a higher proportion of low fifth minute Apgar scores, orotracheal intubation at birth, need for ventilatory support, surfactant use, intraventricular hemorrhage, and other complications. The respiratory distress rates were similar in both groups. Neonatal hypoglycemia, an expected complication of diabetes, was more frequent in the Diabetes Group.

Less spontaneous PTB was observed at the later gestational ages, which could be an effect of obesity and its relation to a more therapeutic rather than spontaneous onset of labor.

Our study has a few limitations. The EMIP was designed to evaluate PTB in Brazil and did not specifically assess the effect of diabetes on maternal and perinatal outcomes. The diagnosis of diabetes was based on data from medical charts and the criteria considered depended on each hospital’s protocol, considering that no specific procedures for diabetes screening were implemented in the study. Furthermore, data regarding gestational age at diagnosis of diabetes, dietary approach, or drugs used to treat diabetes were not available, nor was information on glycemic control, which could have an important impact on pregnancy-related outcomes. Another concern is that the EMIP only included reference centers for high-risk pregnancies in Brazil, which can increase the occurrence of complicated pregnancies. However, this study included a large number of women and representative centers in the majority of Brazilian geographic regions. To the best of our knowledge, this is the only multicenter study in Brazil that has specifically assessed PTB.

## CONCLUSION

Brazilian women with diabetes who had preterm birth were older, more obese, and had a greater proportion of late preterm deliveries, usually by cesarean section as compared to the women without diabetes. They also had a greater frequency of fetal morbidities, such as malformations and polyhydramnios, and a greater proportion of large-for-gestational-age and macrosomic newborn babies.
